# Feasibility and Acceptability of Transcendental Meditation for Indian Healthcare Professionals in a Tertiary Care Hospital: A Pilot Randomized Controlled Feasibility Trial

**DOI:** 10.7759/cureus.105263

**Published:** 2026-03-15

**Authors:** Shruti Niraj, G Niraj, Rajesh Naithani

**Affiliations:** 1 Psychology and Palliative Care, Sri Madhusudan Sai Institute of Medical Sciences and Research, Chikkaballapur, IND; 2 Pain Medicine and Anesthesia, Sri Madhusudan Sai Institute of Medical Sciences and Research, Chikkaballapur, IND; 3 Biochemistry, Graphic Era University, Dehradun, IND

**Keywords:** anxiety, burnout, indian healthcare professionals, perceived stress, transcendental meditation

## Abstract

Healthcare professionals in India are vulnerable to experiencing a high level of burnout, which has consequences for individuals and organizations. This prospective pilot randomized controlled feasibility study examined, for the first time, the feasibility and acceptability of practicing the Transcendental Meditation (TM) technique among healthcare professionals in India. A total of 76 healthcare professionals were randomized to either a TM intervention group or a control group using a stratified randomization method. The feasibility and acceptability measures were designed to assess whether the practice of TM was feasible and acceptable. Secondary outcomes were assessed using validated questionnaires, including the Generalized Anxiety Disorder-7 (GAD-7), the Perceived Stress Scale-4 (PSS-4), the Copenhagen Burnout Inventory (CBI), and the Short Warwick-Edinburgh Mental Well-Being Scale (SWEMWBS). Outcome measures were collected at baseline and 12 weeks post-intervention.

Feasibility and acceptability outcomes indicated good engagement with the intervention. All participants assigned to the TM group attended the individual TM instruction session, and 34 of 38 participants (90%) attended at least four of the five follow-up sessions. Approximately two-thirds of participants reported that practicing TM was feasible or very feasible, and the overall self-reported adherence to TM practice was 79%.

With respect to secondary outcomes, participants in the TM group demonstrated reductions in anxiety and burnout scores and improvements in well-being at the 12-week follow-up compared with the control group. However, no statistically significant change in perceived stress levels was observed, as measured by the PSS-4.

The findings of this pilot randomized controlled feasibility trial demonstrated that TM can be considered as a feasible and acceptable intervention for healthcare professionals in rural India. Preliminary improvements were observed in anxiety, burnout, and well-being. However, these findings should be interpreted cautiously given the exploratory nature of this trial. Substantive studies are recommended to confirm these findings.

## Introduction

Burnout, work-related stress, and anxiety are increasingly recognized as major occupational health concerns among healthcare professionals (HCPs) worldwide. Over the past decade, evidence has documented that burnout affects a substantial proportion of the global healthcare workforce, with approximately 40-60% of physicians reporting at least one symptom of burnout and around one-third of nurses experiencing high levels of emotional exhaustion [1-3].

In India, studies similarly demonstrate a high psychological burden among healthcare workers. A cross-sectional study conducted in 2019 at two tertiary care hospitals in India showed that 51% and 74% of the nursing staff rated experiencing stress and anxiety on the Depression, Anxiety, and Stress Scale (DASS-21) [4]. Approximately two-thirds of the sample, in a study conducted with physicians working at a busy tertiary hospital in North India, reported moderate levels of stress (67.2%), while an additional 13% described high stress levels [5]. Overall, more than 90% of the participants experienced some degree of burnout. This included 16.9% with minimal signs of burnout, 44.3% were at risk of developing burnout, 13.5% demonstrated a severe risk, and 16.6% exhibited a very severe risk of burnout [5]. This study also showed that approximately 30% of medical doctors experienced a moderate to severe level of depression and 17% of those reported suicidal ideations [5].

Burnout results from chronic workplace stress that has not been addressed and managed successfully [6,7]. A recent systematic review reported that the prevalence of burnout in HCPs in India ranged from 23% to 27% [8]. A cross-sectional study with a sample of 100 staff nurses at a tertiary care hospital in South India showed that 86% of the nurses had a high to very high level of burnout and 14% had a moderate level of burnout [9]. This study, however, developed its own self-report questionnaire for this study. Another cross-sectional study used a validated gold-standard questionnaire, the Maslach Burnout Inventory (MBI), in 100 staff nurses at a tertiary care hospital in North India [10]. The result of this study demonstrated a high level of burnout in 33% of participants in the depersonalization domain and 28% in the personal accomplishment domain, respectively. A medium level of burnout was found in 37%, 46%, and 25% of participants in the emotional exhaustion, depersonalization, and personal accomplishment domains, respectively [10].

Probable causes for increased stress, anxiety, and burnout include heavy workload, long working hours, disturbed sleep-wake cycle, and moral injury due to the inability to meet patient needs and demands [8,9,11]. Chronic work-related stress and burnout are significant contributors to impaired job performance, increased absenteeism, reduced productivity, and compromised patient care, with far-reaching consequences for healthcare organizations [9,12]. Conversely, supporting employee well-being improves job satisfaction, enhances performance, strengthens patient care, and reduces staff turnover [12,13]. It is therefore imperative that healthcare organizations actively prioritize the early identification and intervention of workplace stress, alongside initiatives to foster employee well-being. Although structural and organizational changes are essential, effective individual-level strategies are also required to prevent and manage workplace stress.

Mindfulness-based interventions have been shown to be effective in reducing stress and burnout in HCPs [14]. Among the techniques used for stress reduction, a substantial body of research has explored the impact of Transcendental Meditation (TM) on mental, physical, and social well-being. These studies have been conducted in diverse participant groups such as clinicians, students, and veterans, representing a broad spectrum of ages, cultures, and religious affiliations. TM has been described as a natural, simple, and effortless technique practiced for 20 minutes twice each day while sitting comfortably with the eyes closed [15]. Practitioners are instructed to silently repeat a single mantra, a sound without inherent meaning, without engaging in concentration or contemplation; this enables mental activity to naturally quiet down, allowing a state of deep relaxation in both mind and body [16,17]. TM is taught in a structured program and aims to reduce stress and enhance physiological as well as psychological well-being like other mindfulness-based programs. TM is distinguished by its ability to facilitate automatic self-transcending, a process that occurs with minimal cognitive control, unlike other forms of meditation that emphasize focused attention or open monitoring [18]. Over the past few years, clinical trials have demonstrated the efficacy of TM in the reduction of stress and burnout among nurses, physicians, and emergency department clinicians [12,19-21].

Given the high prevalence of work-related stress, anxiety, and burnout among HCPs in India, there is an urgent need for effective and scalable interventions. Despite promising outcomes in international cohorts, scarce data exist on TM practice in Indian HCPs, particularly in resource-constrained and high-demand rural hospitals. Understanding whether this approach can be practically implemented, well-received, and sustained among HCPs in such settings is a crucial step before progressing to large-scale evaluations of effectiveness. The present pilot study was designed to evaluate the feasibility and acceptability of a TM intervention among HCPs in rural India. The primary objective was to assess whether engagement in the TM intervention was feasible and acceptable in this cohort. The secondary objective was to explore the potential effects of TM on psychological outcomes, including anxiety, perceived stress, burnout, and overall well-being. We hypothesized that participation in the TM intervention would be feasible and acceptable for HCPs and that it might be associated with improvements in anxiety, perceived stress, burnout, and well-being.

## Materials and methods

Study design and participants

**Figure 1:**
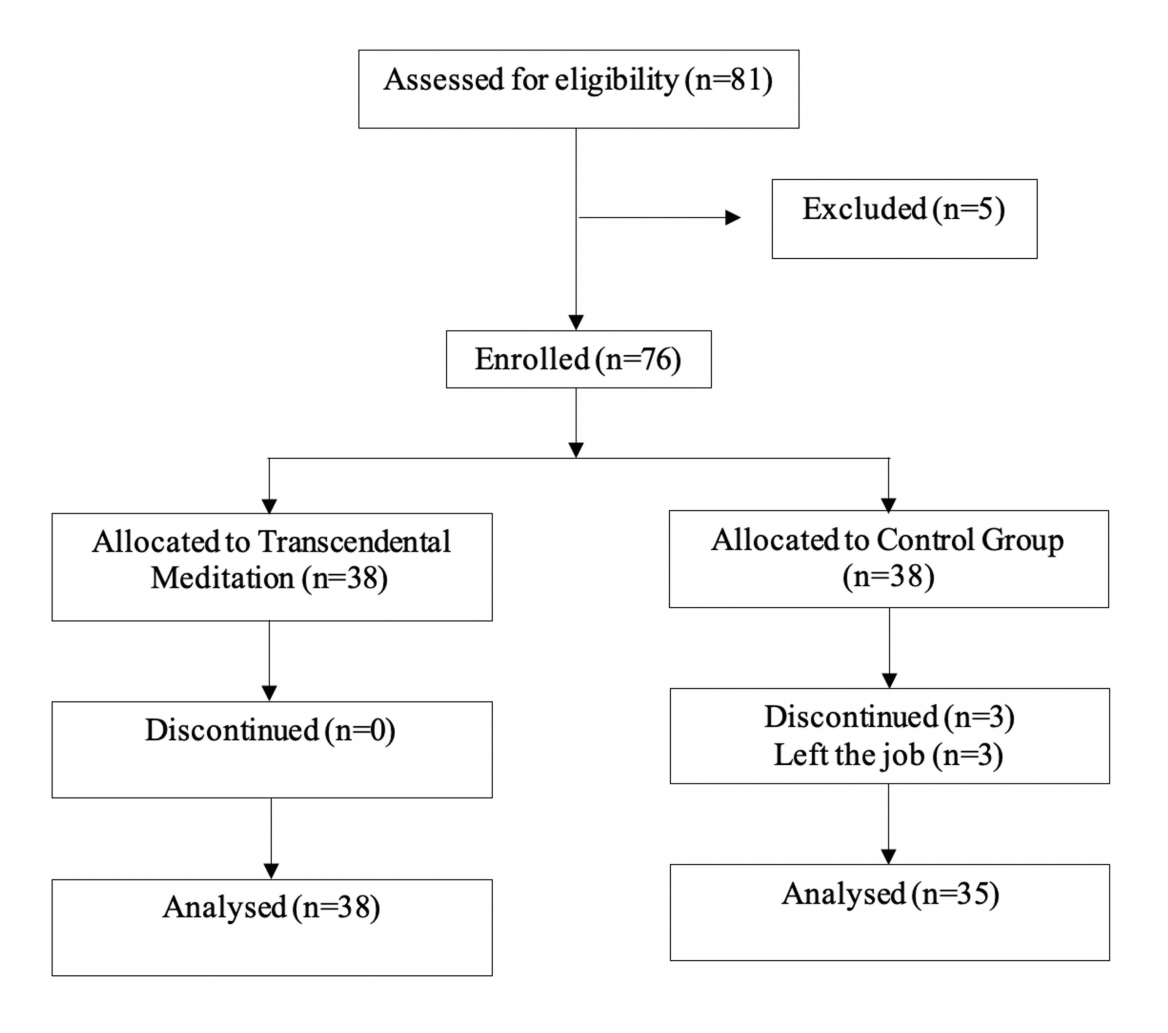
CONSORT trial flowchart CONSORT: Consolidated Standards of Reporting Trials

This was a single-center, open-label, prospective, pilot randomized controlled feasibility trial conducted between August 2024 and January 2025. Eligible participants were randomized using a stratified randomization method (Figure 1). The study commenced after obtaining due approval from the Institutional Ethics Committee of Sri Sathya Sai University for Human Excellence (approval number: IEC/Certificate/17/2024). The study was registered with the Clinical Trial Registry of India (CTRI number: CTRI/2024/07/071645).

The study population consisted of HCPs, including doctors, nursing staff, and frontline allied health staff working at Sri Madhusudan Sai Institute of Medical Sciences and Research, a busy tertiary care hospital located in Chikkaballapur, India (Table 1). A brochure containing the study procedures and contact details of the investigators was sent to all staff via email. The study brochures were also circulated within the social media groups shared by HCPs. Interested participants were contacted and briefed on the study procedures.

**Table 1:** Demographics and baseline characteristics of the participants TM: Transcendental Meditation

	TM group (n=38)	Control group (n=38)
Age (years): mean (SD)	40 (13.4)	41 (12.6)
Gender: M:F (%)	52:47	45:55
Profession: doctor:allied (%)	69:31	73:27
Marital status		
Married (%)	28 (73.7)	26 (68.4)
Single (%)	10 (26.3)	11 (28.9)
Separated (%)	0	1 (2.6)
Years of work experience		
Less than 5	11 (28.9)	21 (55.3)
5-10	8 (21.1)	7 (18.4)
10-15	3 (7.9)	3 (7.9)
More than 15	16 (42.1)	7 (18.4)

HCPs aged 18 years or older, employed on a full-time basis, willing to dedicate time to learn and practice TM, and able to complete both baseline and post-intervention outcome assessments were included in the study. In contrast, exclusion criteria were the following: a history of prior training or initiation in the TM technique, uncontrolled hypertension, unstable psychiatric conditions, and current use of beta-blocker medications.

All participants provided informed written consent to participate in the study. Thereafter, they completed an electronic questionnaire that was created in Google Forms (Google, Alphabet Inc., Mountain View, California, United States), which assessed demographic characteristics, feasibility and acceptability of the intervention, and validated self-reported measures of anxiety, perceived stress, burnout, and well-being. All the instructions and assessments were delivered in the English language. HCPs were not provided remuneration for participation in the study.

Randomization

Participants were randomized utilizing a simple stratified randomization based on age (<40 years vs. ≥40 years), gender (male vs. female), and medical profession (doctors vs. nurses) into the two groups. Eligible participants were randomized on a 1:1 ratio to receive a three-month TM intervention (TM group) or usual treatment comprising access to wellness resources (control group).

Intervention details

TM Group

Four certified TM instructors, who had been extensively trained in the teaching of the TM program, conducted the training. Groups of HCPs started and completed training at different time points between August 2024 and January 2025.

The participants from the TM group received face-to-face training for five consecutive days. During the first day, participants were given preparatory and introductory information on TM. On day 2, following a brief personal interview, participants received individual instruction in TM practice. The individual instruction was to silently repeat a specific sound (mantra) for 20 minutes, twice daily. The duration of the second session was approximately 90 minutes. The sessions 3-5 comprised checking in group meetings to verify the correctness of practice and additional knowledge about TM practice. The check-in session included an individual five-minute interaction with the TM instructor on the correct pronunciation of the mantra. In addition, the TM instructor reviewed how the participant initiated and ended the 20-minute TM session. These sessions were approximately 60 minutes long. The participants were instructed to practice TM at home for 20 minutes twice daily for the duration of the study period. Face-to-face follow-up sessions with the TM trainers were offered once a month to review the correctness of practice and support participants' home practice.

Control Group

The participants in the control group were given access to wellness resources for the duration of the study period. The resources included self-help information on relaxation, diet, exercise, and stress management, as well as attending daily prayer sessions at our institute. The participants in both arms continued with their usual routine work activities during the study period.

Outcome measures

The primary outcome was to assess whether engaging in TM intervention was feasible and acceptable for HCPs. The feasibility and acceptability measures were designed based on previous studies conducted in a similar population [12,21]. Feasibility was defined as attending four out of five training sessions by 80% of the cohort. Participation in each training session was recorded by a research nurse. A self-report measure was designed to assess the acceptability. The acceptability was evaluated using this measure with a score of 4 or 5 on a 5-point Likert scale for ≥80% of the sample. The participants' practice of meditation was tracked after the primary instruction period (week 1) through the three-month follow-up using the acceptability self-report measure. Adherence to at-home meditation practice was defined based on previous studies as practicing TM at least once daily for >5 days of the week [12,20,21]. The secondary outcomes included the following validated questionnaires. The outcome measures were completed at baseline and at the three-month follow-up.

Generalized Anxiety Disorder-7 (GAD-7)

This is a brief self-report questionnaire, widely used in clinical practice for screening, diagnosing, monitoring, and measuring anxiety symptoms [22]. It has good validity and reliability (Cronbach's alpha is 0.92).

Perceived Stress Scale-4 (PSS-4)

The four-item PSS-4 is a modified version of the original perceived stress scale developed in 1983 by Cohen et al. [23]. This measure was designed to assess the degree of stress people felt in unpredictable, out-of-control, and overloaded situations. PSS-4 is a validated measure used to evaluate perceived stress, with higher scores indicating greater stress [24]. It has good validity and reliability (Cronbach's alpha is 0.75).

Copenhagen Burnout Inventory (CBI)

The CBI is a 19-item questionnaire measuring three burnout sub-dimensions [25]. The personal burnout scale has six items and measures the degree of physical and psychological fatigue and exhaustion experienced by a person regardless of their participation in the workforce (i.e., a generic burnout scale). The work-related burnout scale has seven items and measures the degree of physical and psychological fatigue related to work. The client-related burnout scale has six items and measures the degree of physical and psychological fatigue experienced by people who work with clients. Each scale ranged from 0 to 100 points, with a mean score of 50 indicating no/low burnout, a mean score of 50-74 indicating moderate burnout, a mean score of 75-99 indicating high burnout, and a score of 100 indicating severe burnout [25]. It has excellent validity and reliability (Cronbach's alpha is >0.9).

Short Warwick-Edinburgh Mental Well-Being Scale (SWEMWBS)

Overall well-being among participants was measured using the shorter version of the Warwick-Edinburgh Mental Well-Being Scale (WEMWBS) [26]. It is a seven-item scale consisting of questions covering various aspects of well-being. The WEMWBS has shown strong internal reliability in a sample of the general population (Cronbach's α ranges from 0.89 to 0.91) [26]. Items in SWEMWBS are worded positively, and responses are organized on a 5-point Likert-type scale ranging from 1 (none of the time) to 5 (all the time). The SWEMWBS is scored by first summing the scores for each of the seven items, which are scored from 1 to 5. The total raw scores are then transformed into metric scores using the SWEMWBS conversion table [27]. The cumulative score ranges from 7 to 35, and higher scores indicate higher positive well-being. It has good validity and reliability (Cronbach's alpha is >0.8).

Procedures

Following the completion of baseline outcome questionnaires, participants were randomly allocated to either the TM group or the control group. The participants were asked to complete the questionnaires during a three-month follow-up period. The confidentiality of the participants was maintained, and no personal information was disclosed.

Statistical analysis

Statistical analyses were performed using IBM SPSS Statistics for Windows, Version 25.0 (IBM Corp., Armonk, New York, United States). Frequencies and percentages were calculated for the categorical data (demographics, attendance, and other self-report measures of feasibility and acceptance). The mean scores and standard deviation or median and interquartile range were calculated for the continuous data. This study employed a randomized controlled trial design, with participants randomly allocated to the intervention and control groups. Data distribution for each outcome variable was assessed prior to analysis. Normality was evaluated using the Shapiro-Wilk test. A p-value of <0.05 indicated deviation from normality. Variables that met assumptions of normality were analyzed using parametric statistical tests, while non-parametric tests were applied to variables that violated these assumptions. This approach was adopted to ensure the appropriateness and robustness of the statistical analyses while preserving the integrity of the randomized design. 

Changes within each group from baseline to post-intervention were evaluated using paired t-tests for the normally distributed data and the Wilcoxon signed-rank tests for the non-normally distributed data. Depending upon the distribution of the data, either an independent t-test or the Mann-Whitney U test was used to compare the two groups on changes in each outcome measure from baseline to the post-test measurements to explore the impact of TM on burnout, anxiety, perceived stress, and well-being. The significance level (alpha) was set at 0.05. 

## Results

A total of 81 HCPs were screened, out of which 76 were recruited (38 participants in each arm). All 76 participants completed the baseline assessment. Three participants from the control group were lost to follow-up before the 12-week assessment due to leaving their employment during the study period, resulting in a final sample of 73 participants for the post-intervention analysis.

Feasibility and acceptability

**Figure 2:**
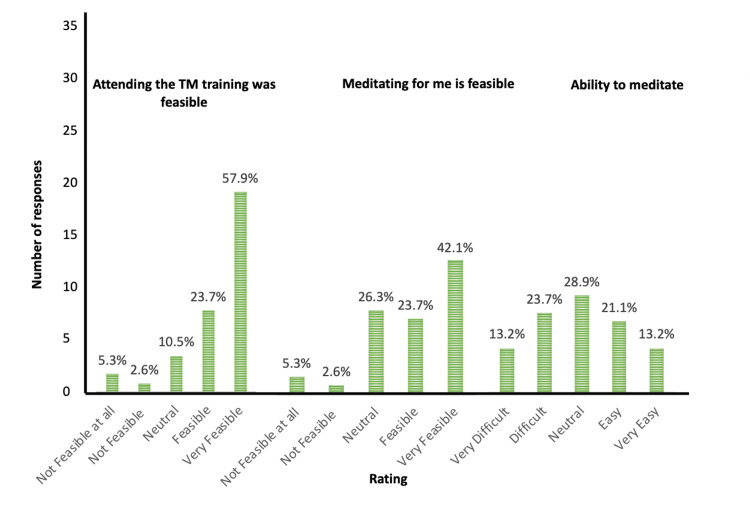
Feasibility of practicing TM TM: Transcendental Meditation

The HCPs found TM training feasible (Table 2). Of the 38 participants recruited, 34 (89.5%) completed at least four out of five sessions. Four participants (10.5%) were able to attend three out of five sessions. The reason for non-attendance was unanticipated emergency on-call work (4/4). All participants (100%) attended the second session where they were provided one-to-one personal instruction in TM. Of the 38 participants, 66% (25/38) reported TM practice was feasible or very feasible. Adherence to TM, identified as practicing at least once daily on most days of the month, was 79% (30/38) (Figure 2).

**Table 2:** Feasibility and acceptability outcomes for TM training for the TM group TM: Transcendental Meditation

	n (%)
Session completion rate (n=38)	
More than 4/5 sessions	34 (89.5)
Less than 3/5 sessions	4 (10.5)
Self-reported meditation practice (n=38)	
Days per week	
0-2 days	4 (10.5)
3-4 days	15 (39.5)
5-7 days	19 (50)
Times per day	
Once per day	30 (78.9)
Twice per day	8 (21.1)
Duration	
0-5 minutes	8 (21.1)
5-10 minutes	6 (15.8)
10-15 minutes	10 (26.3)
15-20 minutes	14 (36.8)
Self-reported feasibility and acceptability (n=38)	
Continue using TM after the completion of the study	
Yes	29 (76.3)
Maybe	7 (18.4)
No	2 (5.3)
Recommend TM to a friend or family	
Yes	37 (97.4)
No	1 (2.6)

**Figure 3:**
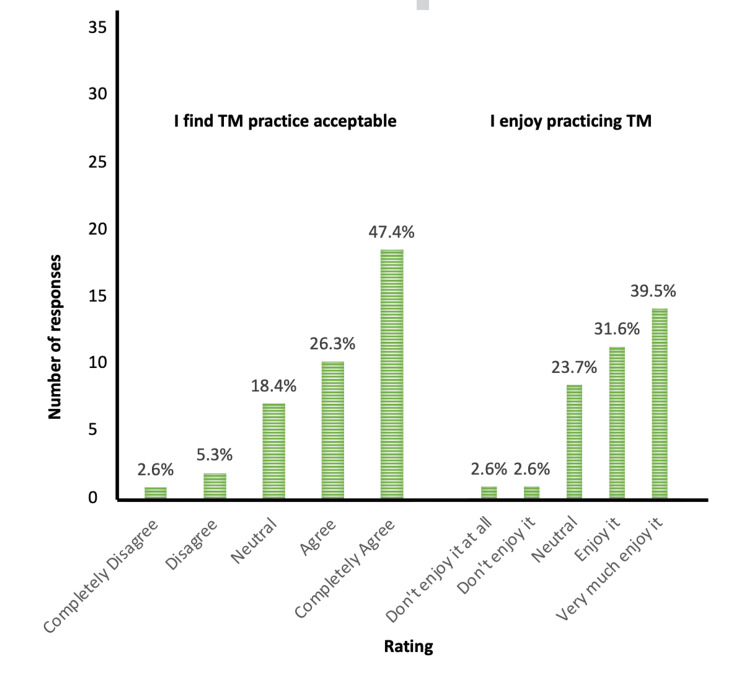
Acceptability of TM TM: Transcendental Meditation

Approximately three-quarters of the sample found TM practice acceptable (28/38), while 71% (27/38) reported enjoying TM practice (Figure 3). Almost all the participants (97.4%) reported that they would recommend TM to a friend or family member.

Intervention effects within the TM group

The questionnaire outcomes for all participants in the TM group at baseline and at 12 weeks are shown in Table 3. There was a statistically significant reduction in anxiety as measured by the GAD-7 questionnaire at 12 weeks post-intervention. In addition, there was a significant improvement in well-being (as measured by SWEMWBS) and personal burnout (as measured by CBI Personal). Although there was a reduction in CBI average scores at 12 weeks, the reduction was not statistically significant.

**Table 3:** Pre- and post-intervention change in questionnaire outcomes for the TM group Summary statistics are mean (95% CI) for parametric tests and mean±standard deviation for non-parametric tests. TM: Transcendental Meditation; GAD-7: Generalized Anxiety Disorder-7; PSS-4: Perceived Stress Scale-4; CBI: Copenhagen Burnout Inventory; SWEMWBS: Short Warwick-Edinburgh Mental Well-Being Scale

Outcome	Timepoint	TM group		Within-group difference		
		n	Mean±SD	Mean (95% CI)	Test statistic	P-value
GAD-7	Baseline	38	4.7±3.7	-	W=80.50	0.003
	12 weeks	38	2.9±2.9			
PSS-4	Baseline	38	6.8±2.8	0.5 (-1.9 to 0.7)	t=1.041	0.305
	12 weeks	38	6.2±2.9			
SWEMWBS	Baseline	38	23.7±5.1	-	W=147.50	0.017
	12 weeks	38	25.7±4.8			
CBI Personal	Baseline	38	40.5±15.5	-	W=168.00	0.015
	12 weeks	38	34.2±12.9			
CBI Work-related	Baseline	38	27.9±19.1	-	W=272.50	0.48
	12 weeks	38	24.4±15.8			
CBI Patient-related	Baseline	38	23.9±20.4	-	W=202.50	0.33
	12 weeks	38	19.5±2.9			
CBI Average	Baseline	38	30.8±15.3	-4.8 (-10.9 to 1.4)	t=1.953	0.058
	12 weeks	38	26.0±11.5			

Intervention effects between groups

Table 4 details the outcome measures between the TM group and the control group at baseline and at 12 weeks post-intervention. The practice of TM resulted in a significant reduction in anxiety, personal burnout, and average burnout scores at 12 weeks compared to participants in the control arm. In addition, when compared to the control intervention, TM practice significantly improved well-being at 12 weeks. 

**Table 4:** Group difference in questionnaire outcomes Summary statistics are mean (95% CI) for parametric tests and mean±standard deviation for non-parametric tests. TM: Transcendental Meditation; GAD-7: Generalized Anxiety Disorder-7; PSS-4: Perceived Stress Scale-4; CBI: Copenhagen Burnout Inventory; SWEMWBS: Short Warwick-Edinburgh Mental Well-Being Scale

Outcome	Timepoint	TM group		Control group		Group difference		
		n	Mean±SD	n	Mean±SD	Mean (95% CI)/mean (SD)	Test statistic	P-value
GAD-7	Baseline	38	4.7±3.7	38	5.5±4.7	4.8 (4.6)	U=355.00	0.001
	12 weeks	38	2.9±2.9	35	6.8±5.3			
PSS-4	Baseline	38	6.8±2.8	38	7.2±2.8	0.5 (-0.89 to 1.86)	t=0.688	0.493
	12 weeks	38	6.2±2.9	35	6.7±3.0			
SWEMWBS	Baseline	38	23.7±5.1	38	22.6±5.1	24.0 (5.4)	U=420.50	0.007
	12 weeks	38	25.7±4.8	35	22.3±5.5			
CBI Personal	Baseline	38	40.5±15.5	38	48.6±21.4	10.3 (1.95 to 18.65)	t=2.458	0.016
	12 weeks	38	34.2±12.9	35	44.5±22.0			
CBI Work-related	Baseline	38	27.9±19.1	38	43.0±18.1	7.2 (-1.62 to 15.92)	t=1.623	0.109
	12 weeks	38	24.4±15.8	35	31.5±21.6			
CBI Patient-related	Baseline	38	23.9±20.4	38	32.2±17.4	21.7 (18.7)	U=617.50	0.595
	12 weeks	38	19.5±2.9	35	23.9±21.5			
CBI Average	Baseline	38	30.8±15.3	38	41.3±14.2	7.3 (0.29 to 14.29)	t=2.078	0.046
	12 weeks	38	26.0±11.5	35	33.3±17.9			

Table 5 details the effect size within groups (pre- and post-intervention). Internal consistency reliability for all measures combined was good for the intervention group (Cronbach's α=0.83) and excellent for the control group (α=0.90).

**Table 5:** Effect size within groups GAD-7: Generalized Anxiety Disorder-7; PSS-4: Perceived Stress Scale-4; CBI: Copenhagen Burnout Inventory; CBI-I: Copenhagen Burnout Inventory subscale Personal burnout; CBI-II: Copenhagen Burnout Inventory subscale Work-related burnout; SWEMWBS: Short Warwick-Edinburgh Mental Well-Being Scale; d: Cohen's D

Measure	Group	Mean difference	SD (difference)	d	Interpretation
PSS-4	Intervention	0.58	3.43	0.17	Small effect
GAD-7	Intervention	1.84	3.43	0.54	Medium effect
Total CBI	Intervention	4.75	9.67	0.49	Medium effect
PSS-4	Control	0.34	1.35	0.25	Small effect
CBI-I	Control	2.51	8.07	0.31	Small to medium effect
CBI-II	Control	12.07	18.18	0.66	Medium to large effect
Total CBI	Control	7.35	12.2	0.6	Medium effect

## Discussion

The practice of TM was feasible and acceptable to Indian HCPs. To the best of our knowledge, this is the first study evaluating the practice of TM in Indian doctors and nurses working in a busy tertiary hospital situated in rural India. All participants attended the one-to-one personal TM instruction session, while 34 (90%) participants were able to attend four out of five sessions. Two-thirds of the participants found practicing TM feasible or very feasible. The self-reported adherence to TM was 79%. This demonstrates that HCPs found practicing TM at least once daily on most days to be a sustainable self-management tool. Nearly three-fourths found practicing TM enjoyable, and almost all reported they would recommend it to their friends and family. These results are comparable to the study completed by Joshi et al. in a sample of emergency clinicians during the COVID-19 pandemic [12]. 

Secondary outcomes

TM practice was found to reduce anxiety as measured on the GAD-7 scale. The mean baseline GAD-7 score in the TM group was 4.76, suggesting mild anxiety. At the three-month follow-up, the mean GAD-7 score was found to be 2.9. The reduction in anxiety symptoms was statistically significant. HCPs who practiced TM reported no statistically significant change in the stress levels as measured by the PSS-4 outcome measure. TM practice was found to enhance overall well-being as measured by the SWEMWBS. The improvement in well-being at the three-month follow-up was statistically significant. HCPs who practiced TM were found to report a reduction in total burnout as measured by the CBI scale at the three-month follow-up. In addition, there was a significant reduction in the subdomain of personal burnout post-intervention. However, work-related burnout and client-related burnout, as measured, did not show any statistically significant change at three months when compared to the baseline scores.

It is interesting to note that the baseline levels of anxiety, stress, and burnout in our HCP cohort were low, while well-being scores were high in both the TM and control arms. Our center is a charitable hospital where both medical education and healthcare are provided completely free of cost. The institute's culture is steeped in a unique mix of social service and spirituality. Working in such an environment could have played a role in mitigating the baseline levels of anxiety, stress, and burnout when compared to hospitals of similar scale in the corporate or state-run sectors. The lower baseline score of anxiety and stress could have had an impact on the reported outcomes at three months following TM practice.

Our hospital provides quality medical treatment to all patients free of charge. This service causes substantial mitigation of distress in patients and their relatives. This is probably reflected in the low baseline work-related and client-related burnout scores. However, personal burnout scores showed a significant reduction in the TM arm. This could suggest that HCPs are prone to personal burnout even in charitable settings. The practice of TM could be recommended to mitigate personal burnout in HCPs.

Strengths

The present study is the first study to demonstrate that TM training and practice are feasible and acceptable for HCPs in a tertiary hospital located in rural India. While preliminary improvements were observed in anxiety, burnout, and well-being, larger studies are required to determine its effectiveness. Finally, the findings provide valuable preliminary data to inform the design of larger randomized controlled trials, including insights related to participant recruitment, retention, and engagement with the intervention.

Limitations

This single-center pilot study was conducted in a small cohort. As a pilot feasibility trial, the study was not powered to test efficacy outcomes, and findings related to stress, anxiety, burnout, and well-being should be interpreted as exploratory. The study failed to demonstrate a reduction in stress levels in HCPs practicing TM when compared to other published evidence. One of the reasons could be the low baseline scores in the treatment arm. In addition, participants were not screened for secondary outcome measures, including perceived stress and anxiety, as the primary objective was to evaluate whether engaging in a TM intervention was feasible and acceptable. In future studies, the control group should be given a matched intervention (such as relaxation training or stress management training). Although social desirability bias was mitigated by conducting an online survey, it is likely to have affected the reporting of the frequency of TM activity. Additionally, the short follow-up period and reliance on self-reported measures may limit generalizability.

Implications and future directions

The findings of this pilot randomized controlled feasibility trial have several important implications. The observed feasibility and favorable acceptability of the TM intervention indicate that it may be a practical and implementable strategy to support the mental well-being of HCPs working in rural and resource-constrained environments. Given its non-pharmacological nature and low-resource demands, TM may be well-suited for incorporation into existing workplace wellness initiatives.

From a research standpoint, the successful application of stratified randomization, along with satisfactory recruitment, retention, and participant engagement, demonstrates the practicality of advancing to a larger, fully powered randomized controlled trial. Future investigations should focus on assessing intervention effectiveness over longer follow-up periods, incorporating objective outcome measures, and examining the scalability and long-term sustainability of TM across varied healthcare contexts. 

## Conclusions

To the best of our knowledge, the feasibility and effects of TM have not yet been evaluated in Indian HCPs. This is the first study demonstrating that TM can be considered as a feasible and acceptable tool for HCPs in rural India. Preliminary findings also suggest potential benefits for reducing anxiety and for enhancing well-being; however, these effects should be interpreted cautiously given the exploratory nature of the study. Larger, adequately powered randomized controlled trials are warranted to confirm these findings and evaluate effectiveness.
